# Transcriptome Analysis of Integument Differentially Expressed Genes in the Pigment Mutant (*quail*) during Molting of Silkworm, *Bombyx mori*

**DOI:** 10.1371/journal.pone.0094185

**Published:** 2014-04-09

**Authors:** Hongyi Nie, Chun Liu, Tingcai Cheng, Qiongyan Li, Yuqian Wu, Mengting Zhou, Yinxia Zhang, Qingyou Xia

**Affiliations:** State Key Laboratory of Silkworm Genome Biology, Chongqing, China; the Key Sericultural Laboratory of the Ministry of Agriculture, Southwest University, Chongqing, China; Uppsala University, Sweden

## Abstract

In the silkworm *Bombyx mori*, pigment mutants with diverse body colors have been maintained throughout domestication for about 5000 years. The silkworm larval body color is formed through the mutual interaction of melanin, ommochromes, pteridines and uric acid. These pigments/compounds are synthesized by the cooperative action of various genes and enzymes. Previous reports showed that melanin, ommochrome and pteridine are increased in silkworm *quail* (*q*) mutants. To understand the pigment increase and alterations in pigment synthesis in *q* mutant, transcriptome profiles of the silkworm integument were investigated at 16 h after head capsule slippage in the fourth molt in *q* mutants and wild-type (Dazao). Compared to the wild-type, 1161 genes were differentially expressed in the *q* mutant. Of these modulated genes, 62.4% (725 genes) were upregulated and 37.6% (436 genes) were downregulated in the *q* mutant. The molecular function of differently expressed genes was analyzed by Blast2GO. The results showed that upregulated genes were mainly involved in protein binding, small molecule binding, transferase activity, nucleic acid binding, specific DNA-binding transcription factor activity and chromatin binding, while exclusively down-expressed genes functioned in oxidoreductase activity, cofactor binding, tetrapyrrole binding, peroxidase activity and pigment binding. We focused on genes related to melanin, pteridine and ommochrome biosynthesis; transport of uric acid; and juvenile hormone metabolism because of their importance in integument coloration during molting. This study identified differently expressed genes implicated in silkworm integument formation and pigmentation using silkworm *q* mutant. The results estimated the number and types of genes that drive new integument formation.

## Introduction

In insects, widespread pigmentation is involved in mimicry [1], [2], sexual selection [3], [4], thermoregulation [5], [6], cuticle hardening [7], [8], territoriality [9], immunity [10], [11] and preventing ultraviolet damage [12]. Body coloration mainly comes from melanin, ommochromes and pteridines in insects. The molecular mechanisms of pigment pathways were clarified using mutants such as *vermilion* (*v*), *scarlet* (*st*), *brown* (*bw*), *white* (*w*), *sepia* (*se*) in *Drosophila* [13]–[15]. The silkworm *Bombyx mori* was domesticated more than 5000 years and has multiple color mutants. Color mutations are reported throughout the silkworm life cycle in larvae [16], [17], pupae [18], moths [19], [20] and eggs [21], so they are ideal material for elucidating the molecular mechanism of pigment pathways. The larval body color in silkworm is the result of interactions among melanin in the cuticle, xanthommatin, sepialumazine and uric acid in the epidermis [22]. In silkworms, melanism (*mln*) was used to determine that arylalkylamine-N-acetyl transferase is important in the melanin pathway in Lepidoptera [19]. The mutant gene in the red egg (*re*) mutant suggests that the mutant product might transport cysteine or methionine into pigment granules, since the mechanisms after 3-hydroxykynurenine is incorporated into pigment granules are still largely unknown. If correct, this would be useful in elucidating the mechanism of the late steps of the ommochrome synthesis pathway [23].

**Figure 1:**
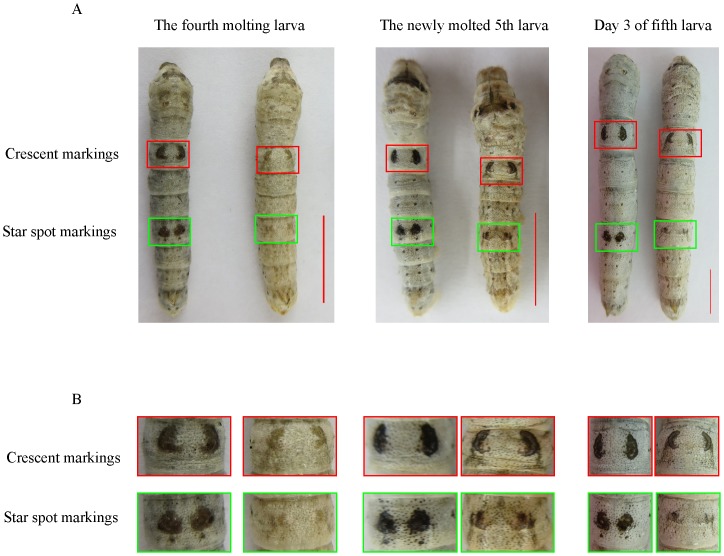
Appearance of *quail* mutant (*q*/*q*) and the wild-type strain (Dazao) at different developmental stages. (A) Right, *q*/*q* mutant; left, wild-type Dazao at fourth molting larval stage, newly molted fifth stage larvae, and day 3 fifth instar insects. Red square, crescent marking; green square, star marking. Bars = 1 cm. (B) Magnified images of larval crescent marking and star spot marking at different developmental stages.

However, these reports mainly focused on single pathway, the relationship of melanin, ommochrome and pteridine pathway is very little. It was reported that melanin, ommochrome and pteridine biosynthesis are increased in *q* mutant [22]. Thus, it is a good model for studying the interaction of different pigments. The *quail* (*q*) mutant has light crescent marking and star spot marking by melanin along the head-to-tail axis, with the star spot marking more dispersive than in wild-type (Dazao). The *q* mutant results in more small black spots on the dorsal surface of the integument than in Dazao; the larval body color of *q* mutant is yellowish-brown with light red on the newly molted fifth instar larva that gradually becomes lighter after eating mulberry leaf (Figure 1).

Although some biochemical and molecular studies of *q* mutant have been performed [22], [24], [25], the mechanisms that result in body color are not clear. In this study, we used a transcriptome approach to investigate gene networks involved in cuticle formation and pigment synthesis in silkworms. Since cuticular pigmentation occurs 16–18 h after head capsule slippage (HCS) at the fourth molt in *P. xuthus* and the fourth molting stage is nearly equivalent in *B. mori* and *P. xuthus* [26], we performed a comprehensive transcriptome analysis of the integument at 16 h after HCS of the fourth molt. Our analysis targeted two main classes of genes: 1) differently expressed genes involved in melanin, ommochrome and pteridine biosynthesis, and 2) differently expressed genes that might function in cuticle formation. Our results determined differently expressed genes involved in pigment biosynthesis pathways, transport of uric acid, juvenile hormone (JH) metabolism, chitin metabolism, cuticular proteins and nuclear receptors. These data add our understanding of the relationship of different pigment pathways and the mechanisms underlying cuticle formation in *B. mori*.

## Materials and Methods

### Silkworm strains and tissue collection

Silkworm *q* mutant strains and Dazao were obtained from the Silkworm Gene Bank of Southwest University, Chongqing, China. All larvae were reared on fresh mulberry leaves at 25°C under long-day conditions (16 h light: 8 h dark). Staging of molting period was based on head capsule slippage (HCS) timing. The whole integument was collected on ice from 3 larvae of each strain just at 16 h after HCS in the fourth molt and other tissues were cleaned out in PBS solution. The mixture of three integuments were washed quickly with water, blotted on filter paper, and then stored at −80°C until RNA extraction.

### RNA preparation and Illumina RNA-seq

Total RNA was prepared using TRIzol (Invitrogen) from the mixture of three whole integument of each strain. RNA integrity was analyzed using an Agilent bioanalyzer (Agilent Technologies 2000). mRNAs were purified with the oligo(dT) magnetic beads, fragmented and used to synthesize cDNA following the TruSeq RNA Sample Preparation v2 Guide(Illumina). Sequencing adaptors were ligated to cDNA fragments by PCR amplification. Sequencing raw data were generated using an Illumina HiSeq 2000 system (Illumina, USA). The raw data presented in this publication have been deposited in NCBI Short Read Archive (http://www.ncbi.nlm.nih.gov/sra/) and are accessible through SRA accession number: SRR1177794 and SRR1177795.

### RNA-seq data analysis

The quality analysis of RNA-seq data was determined using a CLC Genomics Workbench 5.5 following the manufacturer's instructions. The silkworm genome sequence was obtained from the SilkDB (http://silkworm.swu.edu.cn/silkdb/). Reads were mapped to the silkworm genome sequence using CLC Genomics Workbench 5.5. Gene expression levels were calculated using the reads per kb per million reads (RPKM) method [27]. P value<0.05, false discovery rate (FDR)≤0.001 and RPKM>5 were thresholds for gene expression. The RPKM of *q* was divided by the RPKM of Dazao at 16 h after HCS, and the ratio was used to determine differentially expressed genes. Ratio higher than 2.0 or lower than 0.5 was the thresholds for prominent changes. Genes with ratios higher than 2.0 were regarded as upregulated; genes with ratios lower than 0.5 were regarded as downregulated. Differently expressed genes were aligned by BLASTp and results were analyzed for mapping, annotation, enzyme code and combined graph using BLAST2GO software (version 2.6.6, http://www.blast2go.org) with default settings.

### Quantitative reverse transcriptase PCR

RNA was isolated using TRIzol (Invitrogen) from the mixture of three whole integument of each strain, purified with absolute alcohol and treated with with DNase. RNA was reverse transcribed using PrimeScript RT reagent kits (RR037A, Takara) following manufacturer's instructions. Quantitative reverse transcriptase PCR (qRT-PCR) was performed using an ABI7500 real-time PCR system (Applied Biosystems) with a two-step reaction protocol of 40 cycles of 94°C for 3 s and 60°C for 30 s, followed by dissociation for quality control. qRT-PCR mixtures was performed in 15 μL with 1.5 μL of 2 μM specific primers, 7.5 μL SYBR Premix Ex Tag II (Tli RNaseH Plus, Takara) (2×), 0.3 μL ROX Reference Dye II (50×) and 4 μL cDNA. mRNA quantity of each gene was calculated with the 2^−△△Ct^ method [28] and normalized to the silkworm housekeeping gene, ribosomal protein L3 (RPL3, BGIBMGA013567). Ratios after normalization were expressed as fold-change compared with samples from the Dazao integument. The primers are listed in Table S1.

## Results and Discussion

### General transcription patterns

Whole genome mRNA sequencing was used to monitor global changes in gene expression in *q* mutant and Dazao. RNA-seq was performed on integument samples at 16 h after HCS. Quality analysis indicated good RNA-seq data (Figure S1). A total of 33,815,986 paired-end reads (100 bp) were obtained for Dazao, of which 19,724,505 (58.3%) mapped uniquely to the silkworm genome; In *q* mutant, 33,585,319 (61.3%) of 54,754,452 total paired-end reads matched to the silkworm genome. Based on RPKM method [27], the level of 14,623 annotated silkworm genes were determined (Table S2). Applying a FDR cut-off of 0.1% and P value<0.05 resulted in 2905 genes for analysis. To ensure that genes were expressed in *q* and Dazao, RPKM>5 in *q* or Dazao was applied as a threshold. Genes were considered not expressed when the RPKM value was less than 5 in both *q* and Dazao. Differentially expressed genes between *q* and Dazao were identified by expression ratios higher than 2.0 or lower than 0.5. This resulted in 1161 genes that were differentially expressed: 725 upregulated and 436 downregulated genes in *q* (Table S3).

**Figure 2:**
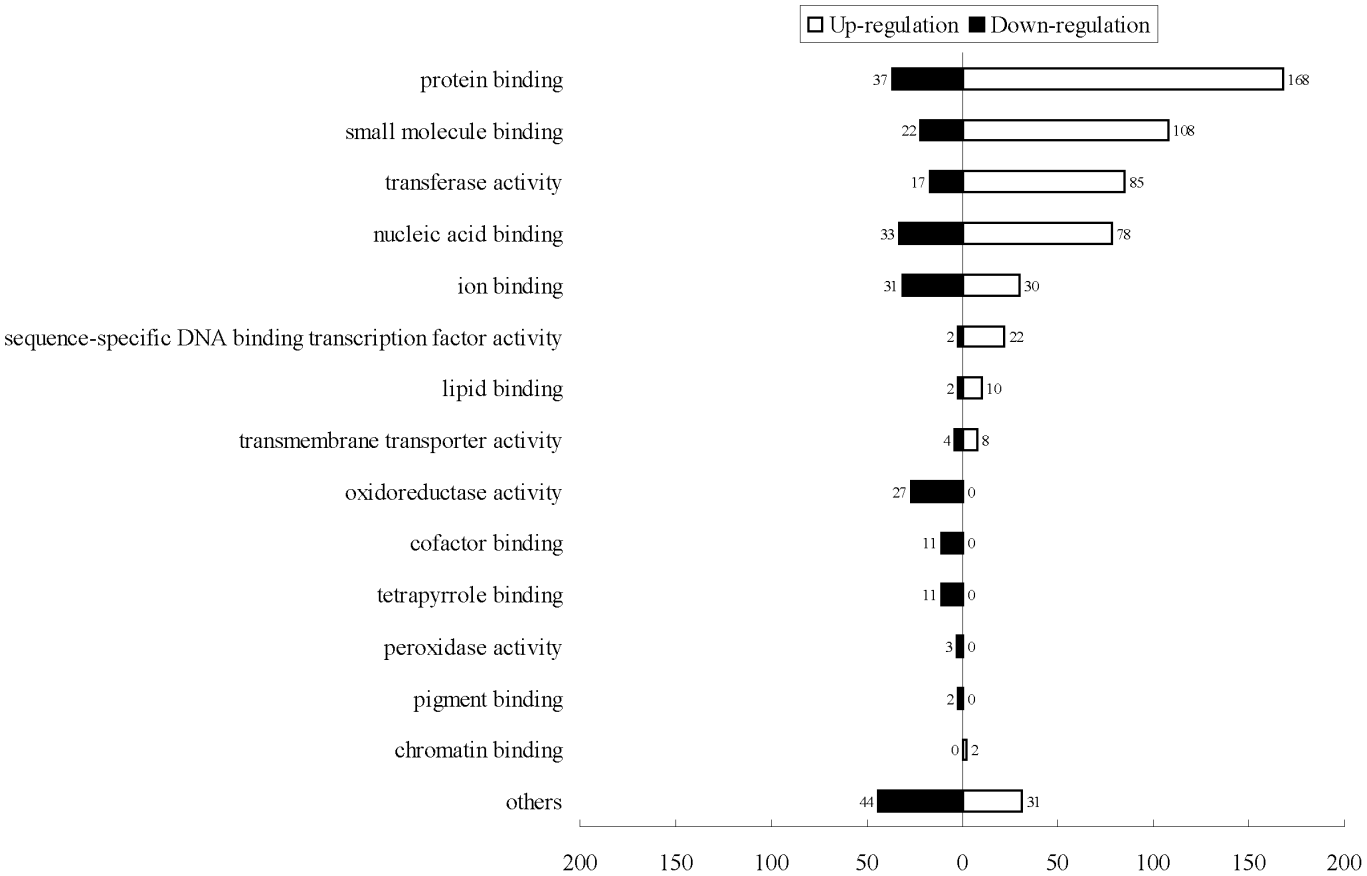
Functional categories of differentially expressed genes in *quail* mutant. Differentially expressed genes were categorized by molecular function. Bars, number of genes. Upregulated or downregulated genes based on Blast2GO analysis were tested for enrichment in specific functional categories.

We analyzed whether transcripts in *q* mutant were enriched in specific molecular functions using Blast2GO (Table S4). Most upregulated gene transcripts in the *q* strain were in protein binding, small molecule binding, transferase activity, nucleic acid binding, specific DNA-binding transcription factor activity, chromatin binding; specific down-regulation of genes in *q* were in oxidoreductase activity, cofactor binding, tetrapyrrole binding, peroxidase activity and pigment binding (Figure 2).

### Validation of data reliability by qRT-PCR

**Figure 3:**
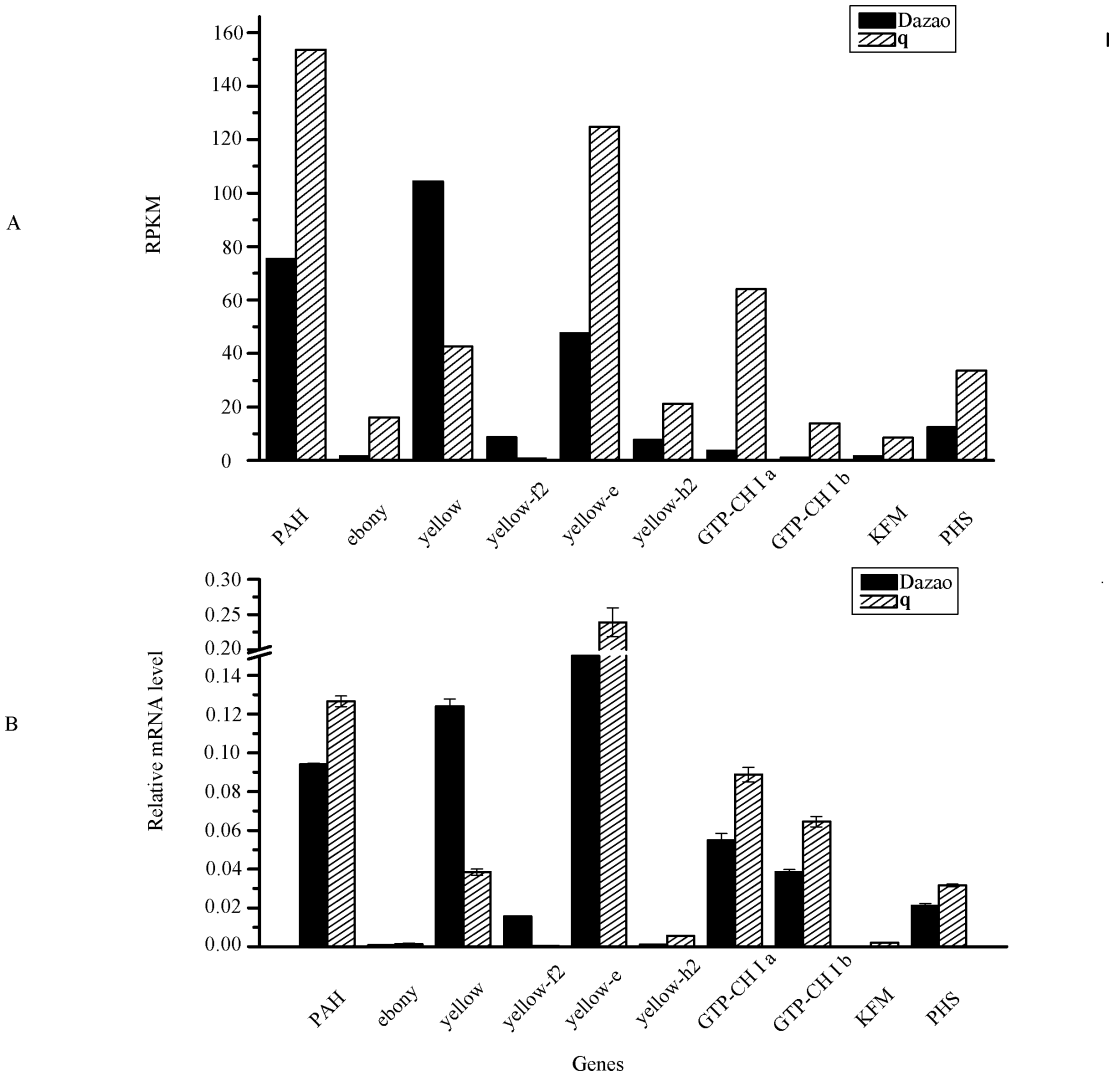
Relative gene expression. (A) Sequencing results. (B) Real-time quantitative PCR results. RPKM, reads per kb per million reads; PAH, phenylalanine hydroxylase; GTPCH I, GTP cyclohydrolase I; KFM, kynurenine formamidase; PHS, phenoxazinone synthetase.

To verify the RNA-seq results, the expression of two silkworm housekeeping genes, RPL3 and glyceraldehyde-3-phosphate dehydrogenase (GAPDH, BGIBMGA007490), were examined in the RNA-seq data. Almost no difference in expression between *q* and Dazao was observed. Previous studies found that the expression of GTP-CH I was higher in *q* mutant [22]. Silkworms have two isforms of GTP-CH I, a and b [18]. The RNA-seq results showed significant upregulation of GTP-CHI a (17.4-fold) and b (12.9-fold) in *q* mutant relative to Dazao, similar to previous studies. To corroborate the RNA-seq results, we selected all differentially expressed genes listed in Table 1 and top ten in up-/down-regulated differentially expressed cuticular protein genes in *q* mutant relative to Dazao for secondary confirmation using qRT-PCR. The similarity of changing trend was 92.6% in Table S5. Figure 3 show that qRT-PCR confirmed the reliability of RNA-seq data. Fold-changes for all genes as determined by qRT-PCR were similar to fold-changes observed by RNA-seq.

**Table 1:** Differentially expressed genes involved pigment pathway, transporting of uric acid, JH metabolism, chitin metabolism and differentially expressed nuclear receptor genes in *q* mutant.

		Fold (*q*/Dazao)		Concordant	
Genes	Name	Fold ^a^	Fold ^b^	up	down
BGIBMGA003866	PAH	2.04	1.34	Yes	
BGIBMGA000031	ebony	9.36	1.83	Yes	
BGIBMGA001149	yellow	0.41	0.31		Yes
BGIBMGA014032	yellow-f2	0.09	0.03		Yes
BGIBMGA010917	yellow-f4	0.09	0.25		Yes
BGIBMGA007253	yellow-e	2.61	1.48	Yes	
BGIBMGA007255	yellow-h2	2.73	5.74	Yes	
BGIBMGA007254	yellow-d	2.04	3.16	Yes	
BGIBMGA003918	yellow-f4-2	3.32	3.27	Yes	
BGIBMGA014224	yellow x	3.15	0.17	No	
BGIBMGA001235	GTP-CH I a	17.40	1.62	Yes	
BGIBMGA008134	GTP-CH I b	12.94	1.67	Yes	
BGIBMGA007856	KFM	5.26	9.79	Yes	
BGIBMGA006740	PHS	2.70	1.49	Yes	
BGIBMGA007285	OBP1	4.95	1.40	Yes	
BGIBMGA007286	OBP2	2.45	2.98	Yes	
BGIBMGA002922	*Bm-w-3*	2.86	3.53	Yes	
BGIBMGA003864	*Bm-os*	2.24	1.50	Yes	
BGIBMGA002581	*Bm-ok*	4.12	2.90	Yes	
BGIBMGA010392	JHAMT	64.51	819.88	Yes	
BGIBMGA000772	JHE	13.26	4.22	Yes	
BGIBMGA013930	JHEH	2.31	2.37	Yes	
BGIBMGA008815	JHDK	2.27	2.37	Yes	
BGIBMGA013971	JH-inducible protein	2.48	1.32	Yes	
BGIBMGA011646	β-N-acetylglucosaminidase	2.27	0.80	No	
BGIBMGA004221	GPI	3.08	3.70	Yes	
BGIBMGA007517	GFAT	4.91	1.86	Yes	
BGIBMGA001609	UDPAP	3.66	1.74	Yes	
BGIBMGA006767	EcR	4.46	1.10	Yes	
BGIBMGA002964	HR38	6.50	1.16	Yes	
BGIBMGA000716	βFTZ-F1	3.02	1.05	Yes	
BGIBMGA007914	HR39	2.99	3.04	Yes	
BGIBMGA007970	E74A	2.37	0.73	No	

**Fold^a^**: Fold change (*q*/Dazao) of gene expression in RPKM.

**Fold^b^**: Fold change (*q*/Dazao) of gene expression in qRT-PCR.

**Abbreviations**: PAH, phenylalanine hydroxylase; GTPCH I, GTP cyclohydrolase I; KFM, kynurenine formamidase; PHS, phenoxazinone synthetase; OBP, Ommochrome-binding protein; JHAMT, JH acid methyltransferase; JHE, JH esterase; JHEH, JH epoxide hydrolase; JHDK, JH diol kinase; GPI, Glucose-6-phosphate isomerase; GFAT, Glutamine:fructose-6-phosphate aminotransferase; UDPAP, UDP-N-acetylglucosamine pyrophosphorylase.

### Differentially expressed genes in melanin synthesis

**Figure 4:**
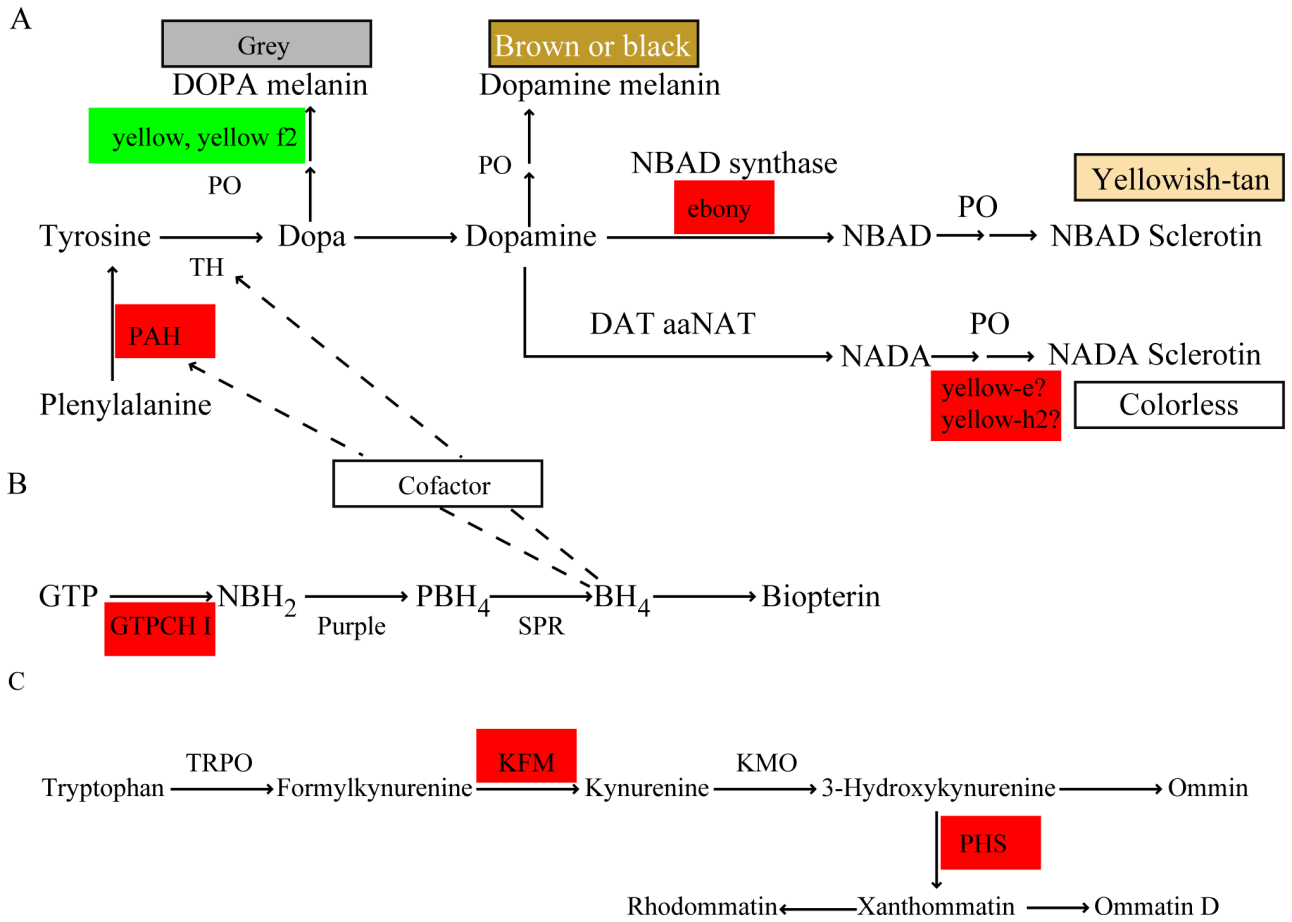
Differentially expressed genes in pigment pathways. (A) Simplified and synthetic melanin; (B) pteridine; (C) ommochrome pathways. Red, upregulated genes; green, downregulated genes in *quail* mutant. PAH, phenylalanine hydroxylase; TH, tyrosine hydroxylase; DAT, dopamine acetyltransferase; DOPA, dihydroxyphenylalanine; NADA, N-acetyl dopamine; NBAD, N-β-alanyl dopamine; PO, phenoloxidases; GTP, guanosine triphosphate; GTPCH I, GTP cyclohydrolase I; NBH_2_, dihydroneopterin triphosphate; BH_4_, tetrahydrobiopterin; TRPO, tryptophan pyrrolase; KFM, kynurenine formamidase; KMO, kynurenine 3-monooxygenase; PHS, phenoxazinone synthetase.

Based on enzymes reported for *D. melanogaster* [29], homologous genes involved in melanin synthesis pathway genes in silkworms were examined in RNA-seq data. Phenylalanine hydroxylase (PAH) was upregulated (2.04-fold, Table 1) and ebony was significantly upregulated (9.36-fold, Table 1), while the expression of yellow, yellow-f2and yellow-f4 decreased (Table 1 and Figure 4A). In addition, yellow relate gene were upregulated in *q* mutant: yellow-e 2.61-fold, yellow-h2 2.73-fold (Table 1). Tyrosine can be obtained from the diet and L-phenylalanine (phenylalanine) generated by PAH for melanin synthesis in insects [30]. With new cuticle synthesized, sclerotization and pigmentation occur during molting, so conversion of phenylalanine to tyrosine by PAH is critical for melanin formation.

Our results suggested that upregulation of PAH provided more tyrosine for melanin formation in *q* mutant. It was reported that the yellow is required to produce black melanin in *Drosophila* and *Bombyx mori* [18], [31]. Previous studies found that yellow-f and yellow-f2 catalyze the conversion of dopachrome into 5,6-dihydroxyindole during dopa melanization [32] and yellow-f4 is similar to *Drosophila* yellow-f [33]. The downregulation of these genes might depress dopa melanization in *q* mutant. During molting, dopamine is involved in the synthesis of light-colored pigments and sclerotization. The ebony gene encodes N-β-alanyl-dopamine (NBAD) synthase, which converts dopamine to NBAD. NBAD is a precursor for the yellow and reddish-brown cuticular pigments of *Drosophila* and *P. xuthus* larvae [31], [34] and is oxidized to NBAD-ortho-quinone which crosslinks cuticle proteins for sclerotization [35]. *Drosophila* ebony mutants have more darkly pigmented thorax and wings [31]; in silkworm ebony mutants, the body color is smoky in the larval and adult stage [18]. It was reported that yellow-e and yellow-h2 may inhibit melanin pigmentation [36]; in silkworm, yellow-e disruption promoted melanin pigmentation in the larval head and tail [37]. Our results suggested that dopa melanization was prevented and dopamine was converted to NBAD or NADA in *q* mutant, resulting in light black crescent markings, light star spot markings and a brown cuticle. In addition, we found that other yellow-related genes (yellow-d, yellow-f4-2 and yellow-x) were upregulated in *q* mutant. The yellow gene family has been identified in many insects, but the function of the gene products is still largely unknown. Three yellow-related genes were upregulated in *q* mutant, so these genes might have other functions in this mutant.

### Differentially expressed genes in pteridine biosynthesis

The genes for enzymes in pteridine biosynthesis were investigated. We found that GTP-CH I was notably upregulated in *q* mutant: GTP-CH I a by 17.40 fold and GTP-CH I b by 12.94 fold (Figure 4B). GTP-CH I is the first enzyme in the pteridine biosynthesis, but both isoforms were upregulated in *q* mutant, suggesting increased conversion of GTP to dihydroneopterin triphosphate compared to wild-type resulting in more BH_4_. GTP-CH I levels are higher in *q* mutant compared to a background strain during the fourth molt [22], consistent with our results. The increase of GTP-CH I in *q* mutant might increase BH_4_ content. Also this was conformed in previous study, which said that the level of BH_4_ was higher in *q* mutant than wild type strain [22]. As BH_4_ is essential as a TH and PAH cofactor during the cuticle screlotization and melanization, the increase of BH_4_ content in *q* mutant might increase PAH and TH catalysis of phenylalanine and tyrosine in the melanin pathway(Figure 4). In *P. xuthus*, GTP-CH I a form is expressed in black regions and is involved in cuticular pigmentation during the fourth molt [2]. Therefore, GTP-CH I a might reinforce melanin synthesis by TH in *q* mutant. Meanwhile, since Dopa melanization might be prevented, it resulted that more dopamine were convert to NBAD or NADA, which increased cuticle sclerotization and brown color.

### Differentially expressed genes in ommochrome biosynthesis

The ommochrome pathway is an important route for eliminating excessive tryptophan metabolites [22] and ommochromes are widely distributed in integument, eyes, testes, wing and extreta in insects [38]. Many mutants with mutations that affect pathway are identified in *Drosophila* and *B. mori*, but the molecular mechanisms of the pathway after 3-hydroxykynurenine is largely unknown. The *q* mutant accumulate high concentrations of xanthommatin in the epidermis [25], so *q* mutant might be affected in later ommochrome pathway steps. Xanthommatin is a yellow-brown pigment derived from condensation of two molecules of 3-hydroxy-kynurenine via phenoxazinone synthetase (PHS) [39]. However, no reports have described the sequence of phenoxazinone synthetase in insects. To identify genes homologous to PHS in *B. mori*, we used the *Streptomyces antibioticus* PHS amino acid sequence (U04283) to query genes predicted in the *B. mori* genome using BLASTP. We found 6 genes homologous to PHS and belonging to the Cu-oxidase family in SilkDB. Among these genes, one was upregulated (2.70-fold, Table 1) in *q* mutant, which might result in increased conversion of 3-hydroxykynurenine to xanthommatin (Figure 4C). In addition, kynurenine formamidase, which converts formylkynurenine into kynurenine, was also upregulated in *q* mutant. Two ommochrome-binding proteins with affinity for phenoxazinone pigments and localized in pigment granules [24] were upregulated in *q* mutant (Table 1). These results suggested that kynurenine formamidase provided more kynurenine, an ommochrome precursor, and upregulated PHS catalyzed 3-hydroxykynurenine to increase xanthommatin resulting in accumulation in *q* mutant.

### Differentially expressed genes in uric acid transport

In addition to melanin, pteridine and ommochrome, uric acid is excreted as a nitrogen metabolite in insects and is indispensable role for white larval epidermis in *B. mori*. Uric acid is synthesized in fat bodies, the midgut and Malpighian tubules [40], transported to the epidermis and accumulates as urate granules, making the silkworm larval skin white and opaque [41]. We found three genes (BGIBMGA002922, BGIBMGA003864 and BGIBMGA002581) involved in transport of uric acid were upregulated (2.86-fold, 2.24-fold and 4.12-fold, Table 1) in *q* mutant. *Bm-w-3* (BGIBMGA002922) is a *w-3^oe^* mutant silkworm gene. Mutants have translucent larval skin from a deficiency in uric acid transport [42]. BGIBMGA003864 is an amino acid transporter of solute carrier proteins involved in uric acid transport in insects and is responsible for the sex-linked translucent (*os*) phenotype [43]. Mutation of an ABC transporter gene (BGIBMGA002581) is responsible for the failure to incorporate uric acid in the epidermis of *ok* mutants of *B. mori* [44]. In addition, *Bm-w-3* is involved in pteridine formation and might transport pteridines in the silkworm [44]. *Bm-w-2* and *Bm-w-3* form a heterodimer responsible for transport of ommochrome precursors. These data suggested that the three genes are involved in transport of uric acid and *Bm-w-3* has important role for transporting pteridine and ommochrome. The expression of these genes was significantly increased in *q* mutant, suggesting that increased uric acid, pteridine and ommochrome were transported to the epidermis and in combination with melanin, resulted in the characteristic body coloration of *q* mutant.

### Differentially expressed genes in juvenile hormone metabolism

Regulation of body coloration by JH is reported in several insects. In swallowtail butterfly, *P. xuthus*, JH regulates larval pattern switches: young caterpillars (from the first to the fourth instars) are mimics of bird droppings, whereas fifth instar larvae have a pattern that camouflages the larvae among host plant leaves [45]. In the tobacco hornworm *Manduca sexta*, the normally black mutant larvae do not turn black when treated with JH during molting [46] because JH prevents ommochrome synthesis [47]. Conversely, JH induces xanthommatin in the silkworm larval epidermis [48]. JH acid methyltransferase (JHAMT) converts JH acids or inactive JH precursors to active JHs in the final step of JH biosynthesis in insects. JHMAT levels correspond to JH biosynthesis rates and JHAMT is detected only in the corpora allata of the silkworm fourth instar larvae [49].

We found that JHAMT was highly expressed in integument of *q* mutant but not in the Dazao strain, which was confirmed by q-PCR (Table S5). Key enzymes (JH esterase, JH epoxide hydrolase and JH diol kinase) (Table 1 and Figure S2) [50], in the JH degradation pathway were also upregulated in *q* mutant. Xanthommatin content is markedly increased after JH injection in silkworms [48]. Therefore, More JH was likely synthesized and degraded in *q* mutant during molting, and might be involved in pigment formation in *q* mutant. A juvenile hormone-inducible protein (BGIBMGA013971) was also upregulated (2.48-fold, Table 1) in *q* mutant.

### Differentially expressed genes in cuticular protein and chitin metabolism pathway

Degradation of old cuticle and regeneration of new cuticle occur during insect molting. The insect cuticle is a thin outer epicuticle with a thicker procuticle of proteins and chitin [51]. In the silkworm, 220 putative cuticular protein genes, expressed mainly in the epidermis and wing disc, have been identified using genome sequences [52]. Of these genes, 62 were differentially expressed in *q* mutant: 15 upregulated and 47 downregulated (Table S6). The expression profiles of related enzymes in chitin metabolism were examined. The results showed that β-N-acetylglucosaminidase, glucose-6-phosphate isomerase, glutamine: fructose-6-phosphate aminotransferase and UDP-N-acetylglucosamine pyrophosphorylase were significantly upregulated in the *q* strain (Table 1). Chitin, a linear polymer of β-(1,4)-N-acetyl-D-glucosamine (GlcNAc), is a structural component of the insect trachea, cuticle, cuticular lining of the foregut, hindgut, and peritrophic membrane that lines the lumen of the midgut [53]. Chitin is digested in the cuticle to GlcNAc in a binary enzyme system of a chitinase and a β-N-acetylglucosaminidase in molting fluid [54]. The chitinase hydrolyzes chitin into oligosaccharides and the β-N-acetylglucosaminidase degrades the oligomers to monomers. β-N-acetylglucosaminidase was upregulated in the *q* mutant, suggesting increased chitin digestion. This result was consistent with degradation of old cuticle. Glucosamine: fructose-6-phosphate aminotransferase (GFAT) is a critical enzyme in chitin synthesis. GFAT is a rate-limiting enzyme and provides the GlcN precursor in chitin biosynthesis [55]. The key enzyme was upregulated in *q* mutant, indicating more chitin in *q* strains. The mechanical properties of cuticle are regulated by factors such as the relative amounts of chitin and proteins, protein composition, and degree of sclerotization [56]. We found that many cuticular proteins were downregulated and some enzymes in chitin synthesis were upregulated in *q* mutant, suggesting higher amounts of chitin and a relatively small amount of cuticular protein than in the Dazao strain. The *q* mutant has more black spots on the dorsal surface of the integument than Dazao, indicating that melanin in the cuticle increased in *q* mutant, facilitating sclerotization.

### Differentially expressed nuclear receptor genes

Nuclear receptors are ligand-regulated transcription factors involved in a variety of biological processes [57]. We surveyed 19 nuclear receptors in the *B. mori* genome [58] in our RNA-seq data. Among them, EcR, βFTZ-F1, HR38 and HR39 were upregulated in *q* mutant (Table 1). Key enzymes in chitin biosynthesis are significantly downregulated and chitin contents in the cuticle are significantly decreased in *Spodoptera exigua* after injection of EcR dsRNA [59], suggesting that EcR is an important regulator in chitin biosynthesis. EcR and key enzymes were upregulated in *q* mutant, which might result in increased chitin. Mutants of βFTZ-F1 show that βFTZ-F1 is necessary for larval molting and is crucial for cuticle formation [60]. Cuticular proteins are expressed in different regions of the *B. mori* epidermis [61]. Three cuticle genes are significantly downregulated in HR38 mutant pupae, disrupting cuticular integrity [62]. These findings indicated HR38 has an important function in cuticle formation. DHR39 is a nuclear receptor with high sequence similarity to FTZ-F1 [57], so DHR39 might have a function similar to FTZ-F1. E74A, an ecdysone-inducible transcriptional factor, was upregulated in *q* mutant. βFTZ-F1 and E74A regulate the promoter of target cuticular protein to determine the time and location of expression [62].

## Supporting Information

Figure S1 Quality analysis of RNA-seq data. (A) Distribution of sequence lengths of *q* and (B) Dazao at 16 h after HCS of fourth molt. Vertical axis, number of sequences. Length normalized to total number of sequences. (C) Distribution of average sequence quality scores for *q* and (D) Dazao at 16 h after HCS of fourth molt. Sequence quality was calculated as the arithmetic mean of its base qualities. Vertical axis, number of sequences at a quality score normalized to total number of sequences. (E) Coverage for the four DNA nucleotides and ambiguous bases of *q* and (F) Dazao at 16 h after HCS of fourth molt. Vertical axis, number of nucleotides per type normalized to total number of nucleotides at that position.
(TIF)

Figure S2 Differentially expressed genes in JH degradation pathway. Red box represents upregulated genes in *quail* mutant.
(TIF)

Table S1 Primer pairs for q-PCR.
(XLS)

Table S2 The RPKM of 14,623 annotated silkworm genes in *q* and Dazao at 16 h after HCS of fourth molt.
(XLS)

Table S3 Differentially expressed genes by RNA-seq at 16 h after HCS in *q* mutant.
(XLS)

Table S4 Functional categories of upregulated and downregulated genes in *q* mutant.
(XLS)

Table S5 Differentially expressed genes were verified by qRT-PCR.
(XLS)

Table S6 Differentially expressed cuticular protein genes.
(XLS)

## References

[1] Rettenmeyer CW (1970) Insect mimicry. Annual review of entomology 15: 43–74.

[2] Futahashi R, Fujiwara H (2006) Expression of one isoform of GTP cyclohydrolase I coincides with the larval black markings of the swallowtail butterfly, *Papilio xuthus* . Insect Biochemistry and Molecular Biology 36: 63–70. 16360951 10.1016/j.ibmb.2005.11.002

[3] Wiernasz DC (1989) Female choice and sexual selection of male wing melanin pattern in Pieris occidentalis (Lepidoptera). Evolution: 1672–1682. 10.1111/j.1558-5646.1989.tb02617.x 28564326

[4] Wiernasz DC (1995) Male choice on the basis of female melanin pattern in *Pieris* butterflies. Animal Behaviour.

[5] Watt WB (1969) Adaptive significance of pigment polymorphisms in *Colias* butterflies, II. Thermoregulation and photoperiodically controlled melanin variation in *Colias eurytheme* . Proceedings of the National Academy of Sciences 63: 767–774. 10.1073/pnas.63.3.767 PMC223518 16591777

[6] Watt WB (1968) Adaptive significance of pigment polymorphisms in *Colias* butterflies. I. Variation of melanin pigment in relation to thermoregulation. Evolution: 437–458. 10.1111/j.1558-5646.1968.tb03985.x 28564757

[7] Sugumaran M (2009) Complexities of cuticular pigmentation in insects. Pigment cell & melanoma research 22: 523–525. 19614888 10.1111/j.1755-148X.2009.00608.x

[8] Sugumaran M (1998) Unified mechanism for sclerotization of insect cuticle. Advances in Insect Physiology 27: 229–334.

[9] Futahashi R, Kurita R, Mano H, Fukatsu T (2012) Redox alters yellow dragonflies into red. Proceedings of the National Academy of Sciences 109: 12626–12631. 10.1073/pnas.1207114109 PMC3412017 22778425

[10] Galko M, Krasnow M (2004) Cellular and genetic analysis of wound healing in *Drosophila* larvae. PLoS Biol 2: e239. 15269788 10.1371/journal.pbio.0020239 PMC479041

[11] Ashida M, Brey PT (1995) Role of the integument in insect defense: pro-phenol oxidase cascade in the cuticular matrix. Proceedings of the National Academy of Sciences 92: 10698–10702. 10.1073/pnas.92.23.10698 PMC40679 11607587

[12] Hu YG, Shen YH, Zhang Z, Shi GQ (2013) Melanin and urate act to prevet ultraviolet damage in the integument of the silkworm, *Bombyx mori* . Archives of insect biochemistry and physiology 83: 41–55. 23575996 10.1002/arch.21096

[13] Ten Have J, Green M, Howells A (1995) Molecular characterization of spontaneous mutations at the *scarlet* locus of Drosophila melanogaster. Molecular and General Genetics MGG 249: 673–681. 8544833 10.1007/BF00418037

[14] Kim J, Suh H, Kim S, Kim K, Ahn C, et al (2006) Identification and characteristics of the structural gene for the *Drosophila* eye colour mutant *sepia*, encoding PDA synthase, a member of the Omega class glutathione S-transferases. Biochemical Journal 398: 451. 16712527 10.1042/BJ20060424 PMC1559464

[15] Mackenzie SM, Howells AJ, Cox GB, Ewart GD (2000) Sub-cellular localisation of the white/scarlet ABC transporter to pigment granule membranes within the compound eye of *Drosophila melanogaster* . Genetica 108: 239–252. 11294610 10.1023/a:1004115718597

[16] Liu C, Yamamoto K, Cheng T-C, Kadono-Okuda K, Narukawa J, et al (2010) Repression of tyrosine hydroxylase is responsible for the sex-linked chocolate mutation of the silkworm, *Bombyx mori* . Proceedings of the National Academy of Sciences 107: 12980–12985. 10.1073/pnas.1001725107 PMC2919899 20615980

[17] Meng Y, Katsuma S, Daimon T, Banno Y, Uchino K, et al (2009) The silkworm mutant *lemon* (*lemon lethal*) is a potential insect model for human sepiapterin reductase deficiency. Journal of Biological Chemistry 284: 11698–11705. 19246455 10.1074/jbc.M900485200 PMC2670173

[18] Futahashi R, Sato J, Meng Y, Okamoto S, Daimon T, et al (2008) *yellow* and *ebony* are the responsible genes for the larval color mutants of the silkworm *Bombyx mori* . Genetics 180: 1995–2005. 18854583 10.1534/genetics.108.096388 PMC2600937

[19] Dai F-y, Qiao L, Tong X-l, Cao C, Chen P, et al (2010) Mutations of an arylalkylamine-N-acetyltransferase, *Bm-iAANAT*, are responsible for silkworm melanism mutant. Journal of Biological Chemistry 285: 19553–19560. 20332088 10.1074/jbc.M109.096743 PMC2885234

[20] Sato K, Matsunaga TM, Futahashi R, Kojima T, Mita K, et al (2008) Positional cloning of a *Bombyx* wingless locus *flügellos* (*fl*) reveals a crucial role for *fringe* that is specific for wing morphogenesis. Genetics 179: 875–885. 18505883 10.1534/genetics.107.082784 PMC2429881

[21] Tatematsu Ki, Yamamoto K, Uchino K, Narukawa J, Iizuka T, et al (2011) Positional cloning of silkworm *white* egg 2 (*w-2*) locus shows functional conservation and diversification of ABC transporters for pigmentation in insects. Genes to Cells 16: 331–342. 21294818 10.1111/j.1365-2443.2011.01490.x

[22] Kato T, Sawada H, Yamamoto T, Mase K, Nakagoshi M (2006) Pigment pattern formation in the *quail* mutant of the silkworm, *Bombyx mori*: parallel increase of pteridine biosynthesis and pigmentation of melanin and ommochromes. Pigment Cell Res 19: 337–345. 16827752 10.1111/j.1600-0749.2006.00316.x

[23] Osanai-Futahashi M, Tatematsu K, Yamamoto K, Narukawa J, Uchino K, et al (2012) Identification of the Bombyx Red Egg Gene Reveals Involvement of a Novel Transporter Family Gene in Late Steps of the Insect Ommochrome Biosynthesis Pathway. Journal of Biological Chemistry 287: 17706–17714. 22474291 10.1074/jbc.M111.321331 PMC3366856

[24] Sawada H, Iino T, Tsusué M (1997) Properties of ommochrome-binding proteins from the pigment granules in epidermal cells of the silkworm, *Bombyx mori* Journal of Sericultural Science of Japan 66..

[25] Ohashi M, Tsusué M, Yoshitake N, Sakate S, Kiguchi K (1983) Epidermal pigments affecting the larval colouration of the silkworm, *Bombyx mori* Journal of Sericultural Science of Japan 52..

[26] Futahashi R, Banno Y, Fujiwara H (2010) Caterpillar color patterns are determined by a two-phase melanin gene prepatterning process: new evidence from tan and laccase2. Evolution & development 12: 157–167. 20433456 10.1111/j.1525-142X.2010.00401.x

[27] Mortazavi A, Williams BA, McCue K, Schaeffer L, Wold B (2008) Mapping and quantifying mammalian transcriptomes by RNA-Seq. Nature methods 5: 621–628. 18516045 10.1038/nmeth.1226 PMC13303166

[28] Livak KJ, Schmittgen TD (2001) Analysis of Relative Gene Expression Data Using Real-Time Quantitative PCR and the 2^-ΔΔCT^ Method. methods 25: 402–408. 11846609 10.1006/meth.2001.1262

[29] True JR (2003) Insect melanism: the molecules matter. Trends in Ecology & Evolution 18: 640–647.

[30] Chen P, Li L, Wang J, Li H, Li Y, et al (2013) BmPAH Catalyzes the Initial Melanin Biosynthetic Step in *Bombyx mori* . PLoS ONE 8: e71984. 23991017 10.1371/journal.pone.0071984 PMC3753331

[31] Wittkopp PJ, True JR, Carroll SB (2002) Reciprocal functions of the *Drosophila* yellow and ebony proteins in the development and evolution of pigment patterns. Development 129: 1849–1858. 11934851 10.1242/dev.129.8.1849

[32] Han Q, Fang J, Ding H, Johnson J, Christensen B, et al (2002) Identification of *Drosophila melanogaster* yellow-f and yellow-f2 proteins as dopachrome-conversion enzymes. Biochem J 368: 333–340. 12164780 10.1042/BJ20020272 PMC1222967

[33] Xia A-H, Zhou Q-X, Yu L-L, Li W-G, Yi Y-Z, et al (2006) Identification and analysis of YELLOW protein family genes in the silkworm, *Bombyx mori* . BMC genomics 7: 195. 16884544 10.1186/1471-2164-7-195 PMC1553450

[34] Futahashi R, Fujiwara H (2005) Melanin-synthesis enzymes coregulate stage-specific larval cuticular markings in the swallowtail butterfly, *Papilio xuthus* . Development genes and evolution 215: 519–529. 16133568 10.1007/s00427-005-0014-y

[35] Andersen SO (2010) Insect cuticular sclerotization: A review. Insect Biochemistry and Molecular Biology 40: 166e178. 19932179 10.1016/j.ibmb.2009.10.007

[36] Futahashi R, Shirataki H, Narita T, Mita K, Fujiwara H (2012) Comprehensive microarray-based analysis for stage-specific larval camouflage pattern-associated genes in the swallowtail butterfly, *Papilio xuthus* . BMC biology 10: 46. 22651552 10.1186/1741-7007-10-46 PMC3386895

[37] Ito K, Katsuma S, Yamamoto K, Kadono-Okuda K, Mita K, et al (2010) Yellow-e determines the color pattern of larval head and tail spots of the silkworm *Bombyx mori* . Journal of Biological Chemistry 285: 5624–5629. 19996320 10.1074/jbc.M109.035741 PMC2820789

[38] Linzen B (1974) The Tryptophan→ Omrnochrome Pathway in Insects. Advances in insect physiology 10: 117–246.

[39] Phillips JP, Forrest HS (1980) Ommochromes and pteridines. In: Ashburmer M, Wright TRF, editors. The Genetics and Biology of *Drosophila*. London, UK: Academic Press.pp. 542–623.

[40] Hayashi Y (1960) Xanthine Dehydrogenase in the Silkworm, Bombyx mori L. Nature 186: 1053–1054. 14400300 10.1038/1861053a0

[41] Kômoto N, Sezutsu H, Yukuhiro K, Banno Y, Fujii H (2003) Mutations of the silkworm molybdenum cofactor sulfurase gene, og, cause translucent larval skin. Insect Biochemistry and Molecular Biology 33: 417–427. 12650690 10.1016/s0965-1748(03)00006-7

[42] Kômoto N, Quan G-X, Sezutsu H, Tamura T (2009) A single-base deletion in an ABC transporter gene causes white eyes, white eggs, and translucent larval skin in the silkworm w-3oe mutant. Insect Biochemistry and Molecular Biology 39: 152–156. 18996197 10.1016/j.ibmb.2008.10.003

[43] Kiuchi T, Banno Y, Katsuma S, Shimada T (2011) Mutations in an amino acid transporter gene are responsible for sex-linked translucent larval skin of the silkworm, Bombyx mori. Insect Biochemistry and Molecular Biology 41: 680–687. 21619931 10.1016/j.ibmb.2011.04.011

[44] Wang L, Kiuchi T, Fujii T, Daimon T, Li M, et al. (2013) Mutation of a novel ABC transporter gene is responsible for the failure to incorporate uric acid in the epidermis of *ok* mutants of the silkworm, *Bombyx mori*. Insect Biochemistry and Molecular Biology. 10.1016/j.ibmb.2013.03.011 23567590

[45] Futahashi R, Fujiwara H (2008) Juvenile hormone regulates butterfly larval pattern switches. Science 319: 1061–1061. 18292334 10.1126/science.1149786

[46] Safranek L, Riddiford LM (1975) The biology of the black larval mutant of the tobacco hornworm, *Manduca sexta* . Journal of Insect Physiology 21: 1931–1938.

[47] Hori M, Riddiford LM (1982) Regulation of ommochrome biosynthesis in the tobacco hornworm, Manduca sexta, by juvenile hormone. Journal of comparative physiology 147: 1–9.

[48] Óhashi M, Tsusué M, Kiguchi K (1983) Juvenile hormone control of larval colouration in the silkworm, *Bombyx mori*: Characterization and determination of epidermal brown colour induced by the hormone. Insect Biochemistry 13: 123–127.

[49] Shinoda T, Itoyama K (2003) Juvenile hormone acid methyltransferase: a key regulatory enzyme for insect metamorphosis. Proceedings of the National Academy of Sciences 100: 11986–11991. 10.1073/pnas.2134232100 PMC218700 14530389

[50] Li S, Zhang Q-R, Xu W-H (2005) Schooley DA (2005) Juvenile hormone diol kinase, a calcium-binding protein with kinase activity, from the silkworm, *Bombyx mori* . Insect Biochemistry and Molecular Biology 35: 1235–1248. 16203205 10.1016/j.ibmb.2005.06.005

[51] Andersen SO, Hojrup P, Roepstorff P (1995) Insect cuticular proteins. Insect biochemistry and molecular biology 25: 153–176. 7711748 10.1016/0965-1748(94)00052-j

[52] Futahashi R, Okamoto S, Kawasaki H, Zhong Y-S, Iwanaga M, et al (2008) Genome-wide identification of cuticular protein genes in the silkworm, *Bombyx mori* . Insect biochemistry and molecular biology 38: 1138–1146. 19280704 10.1016/j.ibmb.2008.05.007

[53] Chen L, Yang W-J, Cong L, Xu K-K, Wang J-J (2013) Molecular Cloning, Characterization and mRNA Expression of a Chitin Synthase 2 Gene from the Oriental Fruit Fly, *Bactrocera dorsalis* (Diptera: Tephritidae). International journal of molecular sciences 14: 17055–17072. 23965972 10.3390/ijms140817055 PMC3759951

[54] Fukamizo T, Kramer KJ (1985) Mechanism of chitin hydrolysis by the binary chitinase system in insect moulting fluid. Insect biochemistry 15: 141–145.

[55] Willis JH, Iconomidou VA, Smith RF, Hamodrakas SJ (2005) Cuticular Proteins. In: Lawrence IG, Kostas I, Sarjeet SG, editors. Comprehensive Molecular Insect Science. Amsterdam: Elsevier.pp. 79–109.

[56] Andersen SO (2005) Cuticular Sclerotization and Tanning. In: Gilbert LI, Iatrou K, Gill SS, editors. Comprehensive Molecular Insect Science. Oxford: Elsevier.pp. 145–165.

[57] King-Jones K, Thummel CS (2005) Nuclear receptors—a perspective from *Drosophila* . Nature Reviews Genetics 6: 311–323. 10.1038/nrg1581 15803199

[58] Cheng D, Xia Q, Duan J, Wei L, Huang C, et al (2008) Nuclear receptors in *Bombyx mori* Insights into genomic structure and developmental expression. Insect biochemistry and molecular biology 38: 1130–1137. 18992339 10.1016/j.ibmb.2008.09.013

[59] Yao Q, Zhang D, Tang B, Chen J, Chen J, et al (2010) Identification of 20-hydroxyecdysone late-response genes in the chitin biosynthesis pathway. PloS one 5: e14058. 21124981 10.1371/journal.pone.0014058 PMC2987807

[60] Yamada M-a, Murata T, Hirose S, Lavorgna G, Suzuki E, et al (2000) Temporally restricted expression of transcription factor betaFTZ-F1: significance for embryogenesis, molting and metamorphosis in *Drosophila melanogaster* . Development 127: 5083–5092. 11060234 10.1242/dev.127.23.5083

[61] Ali MS, Iwanaga M, Kawasaki H (2012) Ecdysone-responsive transcription factors determine the expression region of target cuticular protein genes in the epidermis of *Bombyx mori* . Dev Genes Evol 222: 89–97. 22460818 10.1007/s00427-012-0392-x

[62] Kozlova T, Lam G, Thummel CS (2009) The *Drosophila* DHR38 nuclear receptor is required for adult cuticle integrity at eclosion. Developmental Dynamics 238: 701–707. 19235727 10.1002/dvdy.21860 PMC2845474

