# Supplementary figures and images for: Transcriptome Analysis of Integument Differentially Expressed Genes in the Pigment Mutant (quail) during Molting of Silkworm, Bombyx mori
Source: PLoS One. 2014 Apr 9;9(4):e94185. doi: 10.1371/journal.pone.0094185 (PMC3981777; doi:10.1371/journal.pone.0094185)

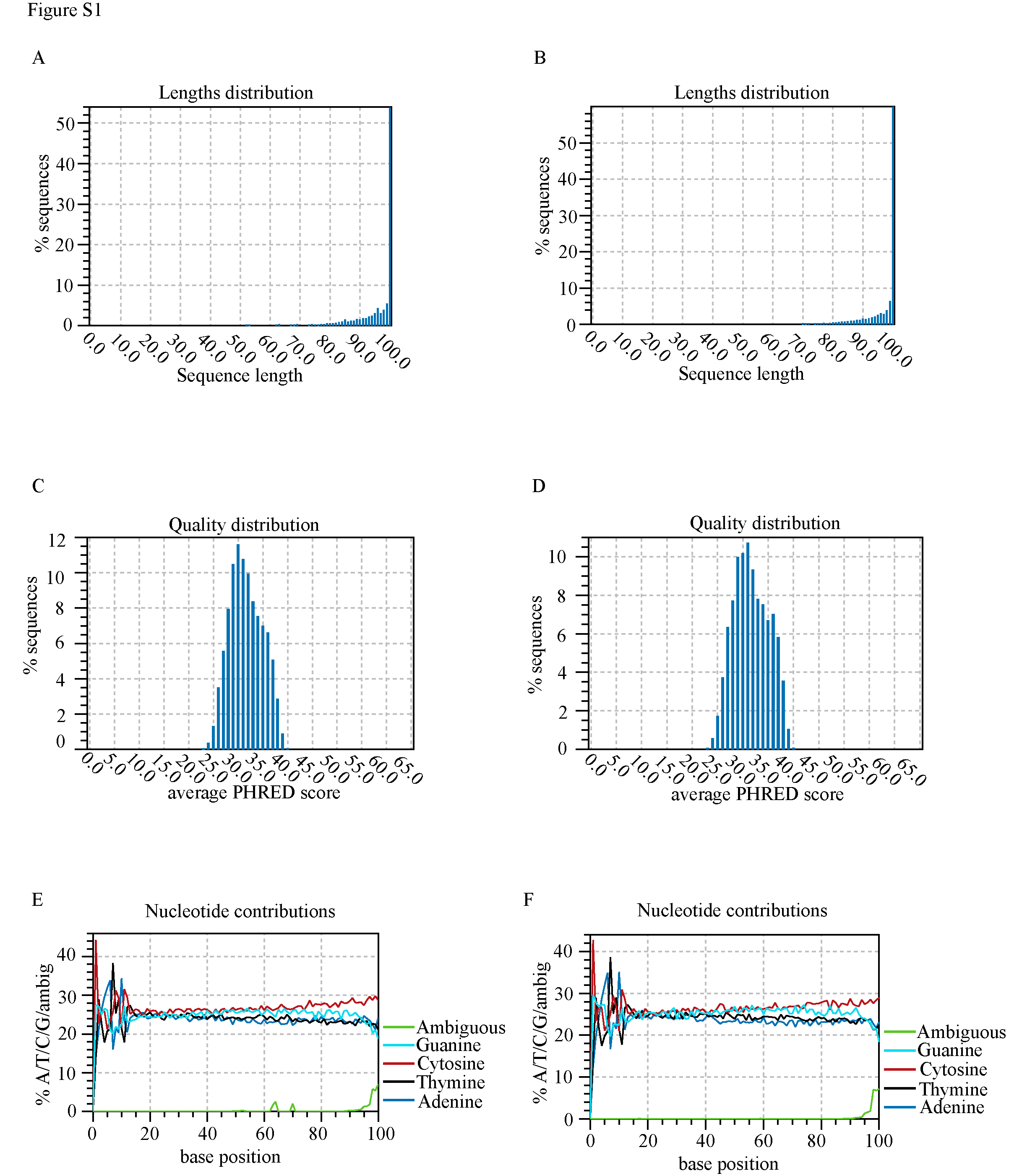

Supplement: Figure S1 — Quality analysis of RNA-seq data. (A) Distribution of sequence lengths of q and (B) Dazao at 16 h after HCS of fourth molt. Vertical axis, number of sequences. Length normalized to total number of sequences. (C) Distribution of average sequence quality scores for q and (D) Dazao at 16 h after HCS of fourth molt. Sequence quality was calculated as the arithmetic mean of its base qualities. Vertical axis, number of sequences at a quality score normalized to total number of sequences. (E) Coverage for the four DNA nucleotides and ambiguous bases of q and (F) Dazao at 16 h after HCS of fourth molt. Vertical axis, number of nucleotides per type normalized to total number of nucleotides at that position. (TIF) [file pone.0094185.s001.tif]

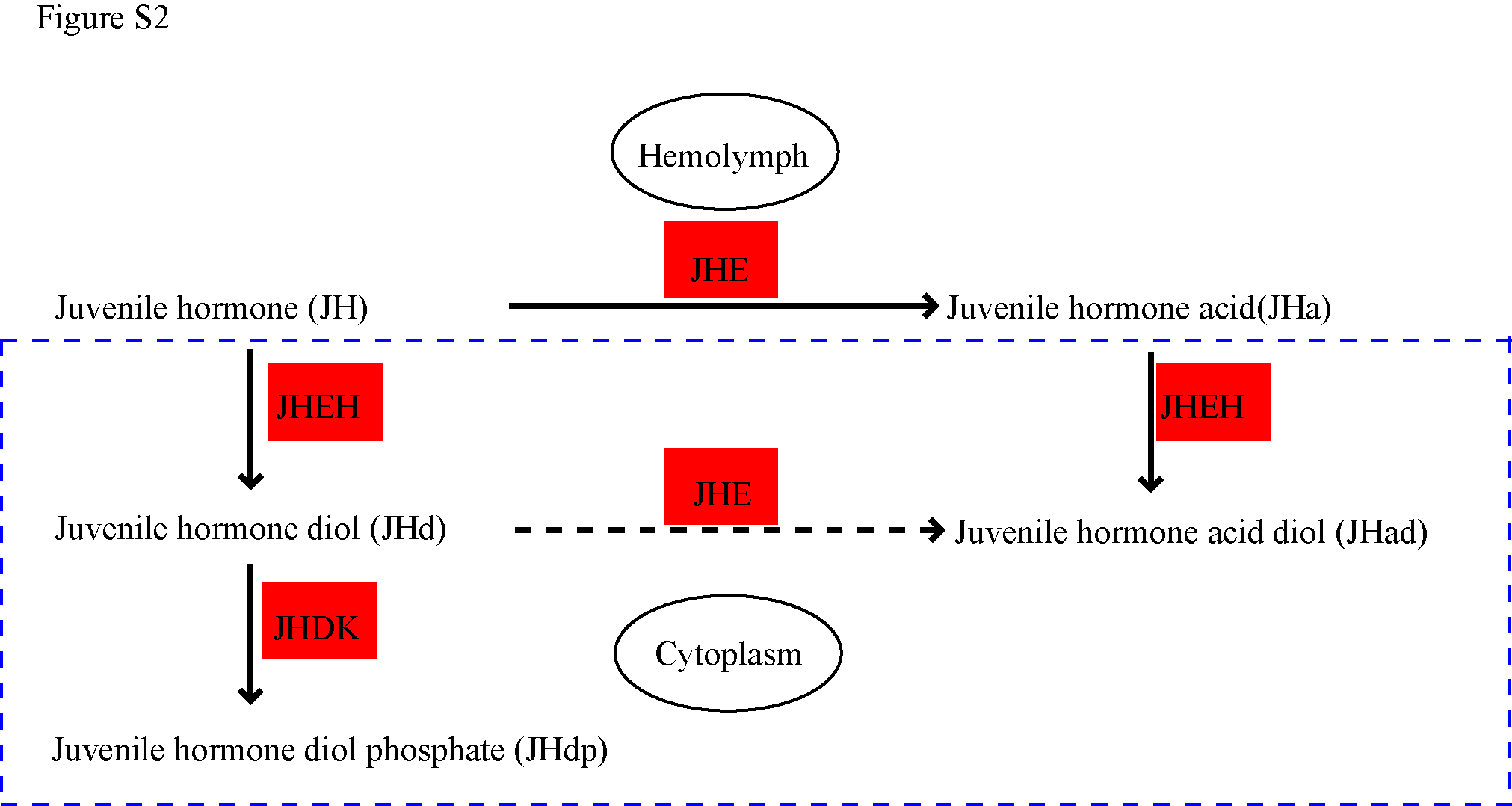

Supplement: Figure S2 — Differentially expressed genes in JH degradation pathway. Red box represents upregulated genes in quail mutant. (TIF) [file pone.0094185.s002.tif]
